# Pediatric Meningococcal Meningitis Complicated by Cerebellar Abscess: A Case Report

**DOI:** 10.7759/cureus.105344

**Published:** 2026-03-16

**Authors:** Khulood Al-Dahash, Ahmad Altelly, Ahmed Attia, Yaser Ali, Rajesh Phatak

**Affiliations:** 1 Academic Affairs, Tawam Hospital, Al Ain, ARE; 2 Academic Affairs, Burjeel Hospital, Abu Dhabi, ARE; 3 Pediatric Intensive Care Unit, Burjeel Hospital, Abu Dhabi, ARE

**Keywords:** brain abscess, case report, cerebellar abscess, complications, meningococcal meningitis, neisseria meningitidis, neuroimaging, pediatric meningitis, vaccination

## Abstract

*Neisseria meningitidis *is a well-recognized cause of bacterial meningitis in infants and adolescents. Its intracranial suppurative complications, such as brain abscess, are exceedingly rare. Only a few published case reports describe this complication, most involving neonates. We report a case of a previously healthy 11-year-old male who presented with a one-day history of acute fever, abdominal pain, vomiting, and irritability. On investigation, cerebrospinal fluid analysis was consistent with bacterial meningitis; polymerase chain reaction detected *Neisseria meningitidis*, confirming meningococcal meningitis. While non-contrast computed tomography was normal, brain magnetic resonance imaging (MRI) demonstrated focal parenchymal changes within the left cerebellar hemisphere, consistent with a cerebellar abscess. Serial MRIs were obtained to follow up the patient’s recovery course. The patient demonstrated marked clinical and radiological improvement following a 21-day course of antimicrobial therapy. Early recognition and prompt neuroimaging are essential in patients with atypical or worsening neurological signs. To the best of our knowledge, this is the first documented case of meningococcal meningitis with cerebellar abscess in an 11-year-old child.

## Introduction

*Neisseria meningitidis* is a major cause of bacterial meningitis in children and adolescents, typically presenting with acute fever, headache, neck stiffness, and altered mental status [1]. While meningococcal infection frequently results in meningitis or septicemia, focal intracranial suppurative complications such as brain abscess are exceedingly uncommon [1,2]. Published estimates suggest that abscess formation occurs in fewer than 1% of invasive meningococcal infections, and only a small number of pediatric cases have been reported in the literature [3-6].

Early diagnosis of brain abscess in the setting of meningitis is clinically challenging. Computed tomography (CT) is often used as the initial imaging modality, but CT may be normal or show only subtle changes in early cerebritis, potentially delaying recognition of an evolving abscess [7]. Magnetic resonance imaging (MRI), particularly with diffusion-weighted sequences, is significantly more sensitive for detecting early parenchymal infection and monitoring lesion progression [8,9].

We describe a rare pediatric case of meningococcal meningitis complicated by MRI-confirmed cerebellar abscess despite a normal CT scan. Serial MRI studies documented the radiologic evolution of the lesion. This case highlights the diagnostic limitations of CT, underscores the importance of early MRI in atypical or persistent presentations, and contributes to the limited literature on meningococcal brain abscess in children.

## Case presentation

An 11-year-old previously healthy male presented with a one-day history of fever, abdominal pain, vomiting, and irritability, with rapid clinical progression. Fever was unresponsive to antipyretics and associated with one episode of non-bilious and non-projectile vomiting and diffuse abdominal pain. There was no sensitivity to light or sound, no cough, no change in bowel habits, no urinary complaints, and no rash. Review of systems was unremarkable except for a mild sore throat and left ear pain, which resolved. Past medical and surgical history was insignificant. No known allergies and not on any medications. Developmental history was appropriate for age. Vaccination history was verified through review of the patient’s vaccination card; all scheduled vaccinations had been received according to the United Arab Emirates (UAE) national immunization schedule for his birth cohort. No history of sick contacts, recent travel, or similar symptoms among family members.

In the hours before presentation, the family noted episodic bizarre behavior lasting five to six minutes with intermittent somnolence. There was no prior history of seizure, weakness, headaches, or altered level of consciousness.

On initial evaluation, the patient appeared acutely unwell, irritable, and intermittently somnolent. The patient was febrile with a tympanic temperature of 39.6 °C, tachycardic with a heart rate of 145 beats per minute, normotensive with a blood pressure of 119/72 mmHg, and had a respiratory rate of 22 breaths per minute. Neurological examination revealed a Glasgow Coma Score (GCS) of 12/15 (E4, V4, M4) with fluctuating levels of consciousness; formal assessment of tone, power, and reflexes was limited by poor cooperation. There was no rash noted. The remainder of the physical examination was unremarkable.

During reassessment in the emergency department, he developed recurrent vomiting and rapid neurological deterioration (GCS 12 to 10), requiring endotracheal intubation and admission to the pediatric intensive care unit (PICU). Repeat vital sign demonstrated a temperature of 36.5 °C, heart rate of 110 beats per minute, and respiratory rate of 18 breaths per minute. Neurological examination in the PICU, performed approximately two to three hours after initial presentation, was conducted under sedation with fentanyl and midazolam. Findings included no focal neurological deficits, bilaterally equal, round, and reactive pupils, with no neck stiffness or photophobia noted. Formal assessment of tone, power, and reflexes was precluded by sedation. 

Labs, including blood cultures, were obtained before initiating antibiotics. He was commenced on hypertonic saline and empirical intravenous antibiotics, including ceftriaxone and vancomycin. A urinary catheter was placed for monitoring. Laboratory evaluation demonstrated markedly elevated C-reactive protein (CRP) and procalcitonin, leukocytosis with neutrophil predominance, metabolic acidosis, stress hyperglycemia, vitamin K deficiency with coagulopathy, and mild hyponatremia (Table 1). The non-contrast CT brain was unremarkable. 

**Table 1:** Laboratory investigations at admission and during the hospital course. ↑, above reference range; ↓, below reference range; -, not performed or not applicable. aPTT, activated partial thromboplastin time; CSF, cerebrospinal fluid; INR, international normalized ratio; PCR, polymerase chain reaction; PT, prothrombin time; RSV, respiratory syncytial virus; WBC, white blood cell count

Parameter	Day 1 (Admission)	Day 2	Day 3	Day 5	Reference range
Inflammatory markers					
C-reactive protein (mg/L)	186 ↑	268 ↑	311 ↑	65.7 ↑	<5
Procalcitonin (ng/mL)	24.10 ↑	31.90 ↑	20.40 ↑	4.77 ↑	<0.05
Complete blood count					
White blood cell count (×10⁹/L)	15.42 ↑	10.39	12.80 ↑	11.06	4.0-11.0
Hemoglobin (g/dL)	12.2	9.2 ↓	9.3 ↓	10.5 ↓	11.5-15.5
Platelets (×10⁹/L)	209	142 ↓	193	268	150-400
Coagulation					
PT (seconds)	15.30 ↑	15.20 ↑	-	11.90	11.0-13.5
INR	1.5 ↑	1.5 ↑	-	1.1	0.8-1.2
aPTT (seconds)	31.5	-	34.6	-	25-35
Vitamin K (ng/mL)	<0.10 ↓	-	-	-	0.10-2.20
Routine chemistry					
Sodium (mmol/L)	133 ↓	141	141	140	135-145
Potassium (mmol/L)	3.6	3.6	3.7	-	3.5-5.1
Bicarbonate (mmol/L)	18 ↓	23	22	23	22-29
Albumin (g/L)	-	34.9 ↓	37.0 ↓	36.4 ↓	35-50
Glucose (mmol/L)	11.40 ↑	-	-	-	3.9-7.8
Lactate (mmol/L)	5.9 ↑	-	-	-	0.5-2.2
Cerebrospinal fluid analysis (Day 1)					
Appearance	Cloudy	-	-	-	Clear
WBC count (cells/μL)	2,300 ↑	-	-	-	0-5
Polymorphs (%)	98	-	-	-	0-6
Predominant cell type	Neutrophils	-	-	-	None
Protein (g/L)	2.23 ↑	-	-	-	0.15-0.45
Glucose (mmol/L)	0.67 ↓	-	-	-	2.5-4.4
PCR: *Neisseria meningitidis*	Detected	-	-	-	Not detected
Microbiology					
Blood culture (×2)	Negative	-	-	-	Negative
CSF culture	Negative	-	-	-	Negative
Urine culture	Negative	-	-	-	Negative
Rapid Group A Strep	Negative	-	-	-	Negative
Influenza A/B antigen	Negative	-	-	-	Negative
RSV antigen	Negative	-	-	-	Negative

Lumbar puncture was performed after correction of coagulopathy with intravenous vitamin K and fresh frozen plasma. Cerebrospinal fluid (CSF) was turbid in appearance with normal opening pressure and marked neutrophilic pleocytosis. Multiplex polymerase chain reaction (PCR) testing using the BioFire FilmArray Meningitis/Encephalitis Panel (BioFire Diagnostics, Salt Lake City, UT) detected *Neisseria meningitidis*, confirming meningococcal meningitis (Table 1). Public health authorities were notified, and chemoprophylaxis was administered to household and close contacts in accordance with public health authority guidance.

**Figure 1:**
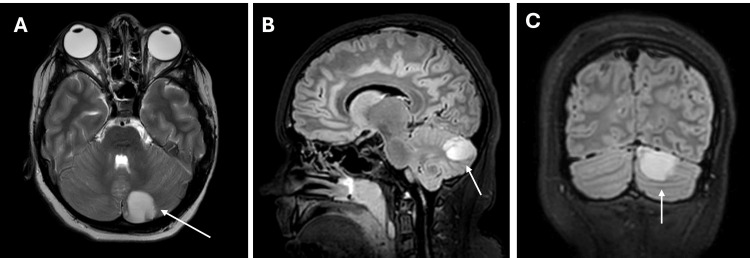
Brain MRI demonstrating a focal lesion in the left cerebellar hemisphere. (A) Axial T2-weighted image demonstrating a hyperintense lesion within the left cerebellar hemisphere (white arrow). (B) Sagittal FLAIR image confirming the posterior fossa location (white arrow). (C) Coronal FLAIR image demonstrating the extent of the signal abnormality (white arrow). No significant mass effect or hydrocephalus is present. FLAIR, fluid-attenuated inversion recovery

By the second day, the patient remained sedated and ventilated. His mean arterial pressure (MAP) was at the lower limit of normal; a small dose of norepinephrine was briefly administered to maintain MAP above 70 mmHg. Given the atypical clinical course, rapid progression, and absence of expected improvement with empiric antimicrobial therapy, there was clinical suspicion of a meningitis-associated intracranial complication warranting further neuroimaging. Despite a normal initial CT scan, a brain MRI with and without contrast was performed and demonstrated focal parenchymal changes within the left cerebellar hemisphere, consistent with a forming cerebellar abscess (Figure 1), without hydrocephalus or mass effect. Multidisciplinary team consultation recommended continuing the same antibiotic regimen and repeating imaging.

The patient demonstrated progressive clinical improvement and was successfully extubated on day three. By day four, vasopressor support was discontinued, and invasive lines and supportive devices were removed, with continued neurological improvement. On day five, he was afebrile, with improving inflammatory markers, and was transferred to the general pediatric ward (Table 1).

**Figure 2:**
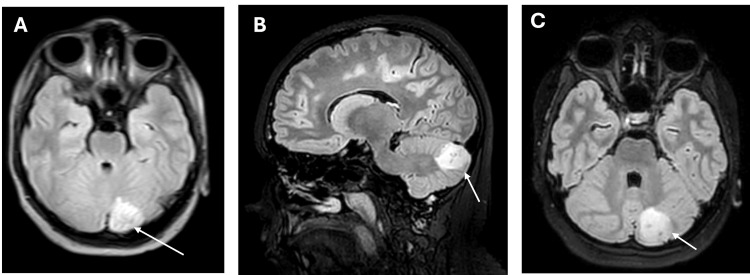
Follow-up brain MRI on day six demonstrating an evolving lesion in the left cerebellar hemisphere. (A) Axial FLAIR image on day six demonstrating interval evolution of the left cerebellar lesion with mild surrounding vasogenic oedema (white arrow). (B) Sagittal FLAIR image confirming the posterior fossa location with no fourth ventricular compression (white arrow). (C) Coronal FLAIR image demonstrating the extent of the evolving lesion (white arrow). No significant mass effect or hydrocephalus is present. FLAIR, fluid-attenuated inversion recovery

A repeat brain MRI on day six demonstrated interval evolution of the left cerebellar abscess with surrounding edema but no mass effect (Figure 2). Blood, urine, and CSF cultures later returned negative. On day seven, the case was reviewed at a multidisciplinary team meeting, which recommended completion of a 21-day course of intravenous ceftriaxone. Although continued inpatient treatment was advised, the patient’s family requested early discharge in view of his clinical stability. After counseling and education, discharge was arranged with home intravenous antibiotic therapy and structured outpatient follow-up.

**Figure 3:**
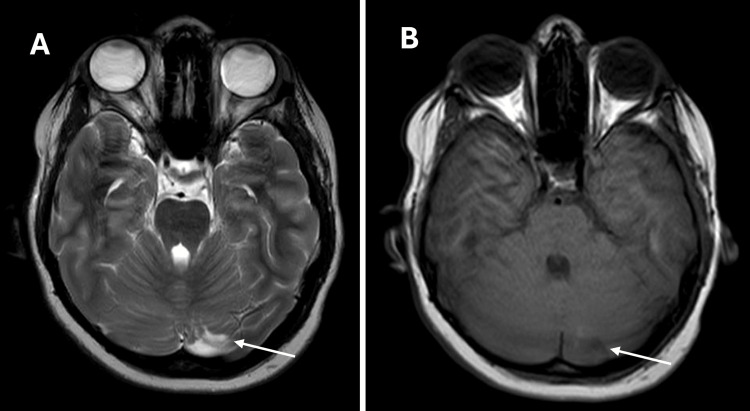
Follow-up non-contrast brain MRI demonstrating interval resolution of the left cerebellar abscess. (A) Axial T2-weighted image demonstrating marked reduction in size of the left cerebellar lesion with near-complete resolution of surrounding signal abnormality (white arrow). (B) Axial T1-weighted image showing no residual pathological signal, consistent with resolving abscess. No mass effect or hydrocephalus is present (white arrow). Contrast administration was declined by the patient's family; post-contrast assessment was, therefore, not performed. MRI, magnetic resonance imaging

Following discharge, the patient was closely monitored through structured outpatient follow-up. At two days post-discharge, he remained clinically well, with no recurrence of fever or neurological symptoms, and intravenous ceftriaxone was continued as planned. He subsequently completed the full intended course of intravenous ceftriaxone with sustained clinical improvement. At 26 days post-discharge, clinical and neurological examination were normal, and follow-up brain MRI demonstrated interval resolution of the cerebellar abscess without concerning features (Figure 3). Findings were reviewed at a multidisciplinary team meeting, and, given the favorable clinical and radiological response, the team consensus was to discontinue antimicrobial therapy with ongoing clinical follow-up recommended. At approximately 10 weeks post-discharge, further outpatient review confirmed continued clinical well-being with no neurological complaints. Post-recovery meningococcal vaccination and formal audiological assessment were recommended at follow-up.

## Discussion

Brain abscesses in children are most commonly caused by *Streptococcus *and *Staphylococcus* species, with posterior fossa involvement accounting for a minority of cases [2,9]. Abscess formation secondary to *Neisseria meningitidis* has been reported only sporadically, predominantly in neonates presenting with multiple supratentorial lesions and significant morbidity [3-6]. The present case is, therefore, notable for demonstrating an isolated cerebellar abscess in an older child, a combination not previously described in the literature.

The pathophysiological mechanisms underlying abscess formation in meningococcal disease are not fully understood. Hypothesized mechanisms include localized cerebritis resulting from an intense inflammatory response, hematogenous bacterial seeding, or impaired host immune regulation during acute infection [5,9]. Early and appropriate antimicrobial therapy is thought to reduce the risk of such complications, which may explain their rarity in the modern antibiotic era.

The absence of a petechial or purpuric rash, a hallmark feature of meningococcal septicemia, further contributed to the diagnostic challenge in this case. While a non-blanching rash is a well-recognized feature of invasive meningococcal disease, it may be absent or non-specific in its early stages, particularly in cases presenting predominantly with meningitis rather than septicemia [10]. This underscores the importance of maintaining clinical vigilance for meningococcal disease even in the absence of cutaneous manifestations.

Diagnosis of brain abscess in the setting of meningitis can be challenging, as early clinical features may overlap with the expected course of severe meningeal inflammation. In this case, the presence of persistent fever and neurological deterioration despite initial therapy prompted further neuroimaging. While the initial non-contrast CT scan was normal, subsequent MRI revealed early cerebellar parenchymal involvement consistent with abscess formation. This highlights the diagnostic limitations of CT, particularly in early cerebritis and posterior fossa pathology, and reinforces the superior sensitivity of MRI, especially diffusion-weighted imaging, for early detection and monitoring of intracranial infections [7,8]. This case reinforces that the threshold for proceeding to MRI should be guided by clinical judgement, particularly when the presenting course is atypical or when neurological status fails to improve as expected despite treatment.

The patient was successfully managed with prolonged intravenous antimicrobial therapy without surgical intervention. Ceftriaxone was selected given its established efficacy against *Neisseria meningitidis* and excellent cerebrospinal fluid penetration, with a 21-day course determined by the multidisciplinary team in view of the intracranial suppurative complication. Surgical drainage was not pursued given the small abscess size, absence of mass effect, and favorable clinical trajectory, consistent with published evidence supporting medical management in clinically stable patients with close radiological monitoring [2,9]. Serial MRI proved invaluable in documenting lesion evolution and guiding the decision to maintain non-operative management and is recommended as standard practice in similar presentations.

This case highlights an important gap in meningococcal vaccine coverage. Review of the patient’s vaccination card confirmed receipt of all scheduled vaccinations for his birth cohort; however, routine meningococcal vaccination was not part of the UAE national immunization program for children of his age group at the time of presentation. Meningococcal conjugate vaccination in the UAE was introduced in 2019, initially targeting adolescents aged 16-18 years, with no routine dose scheduled for younger children [11]. Very recently, the UAE national immunization program was updated in January 2026 to significantly expand meningococcal vaccine coverage, introducing doses at 12 months, 10-11 years, and 16-18 years of age [12]. This case may represent exactly the clinical scenario that underscores the public health rationale for this expanded coverage and illustrates the vulnerability of the pre-adolescent age group that existed under the previous schedule. Post-recovery meningococcal vaccination was recommended.

To the best of our knowledge, this is the first reported case of meningococcal meningitis complicated by cerebellar abscess in an 11-year-old child. This case underscores the importance of maintaining a high index of suspicion for atypical intracranial complications in children with meningitis who exhibit persistent fever, altered neurological status, or an unexpected clinical course, even in the absence of focal neurological deficits.

Several limitations of this report warrant acknowledgment. Meningococcal serogroup determination was not possible, as the BioFire® FilmArray® ME panel identifies *Neisseria meningitidis* at the species level only; serogroup data would have provided additional epidemiological and public health value. Complement assessment was not performed during the acute admission, as the patient had no prior history of recurrent or invasive infections; this is recommended in future similar cases. Regarding audiological follow-up, formal pure tone audiometry was not performed; however, the risk of sensorineural hearing loss following meningococcal meningitis is considerably lower than that associated with pneumococcal meningitis, with published incidence estimates of 10%-24% for *Neisseria meningitidis* compared with up to 31% for *Streptococcus pneumoniae* [13,14,15]. Nevertheless, formal audiological assessment is recommended in future similar cases. Finally, long-term neurodevelopmental and cognitive outcomes could not be fully assessed given the recency of this report, emphasizing the need for extended follow-up.

## Conclusions

Cerebellar abscess due to meningococcal meningitis is exceedingly rare in pediatrics. This report highlights the importance of timely neuroimaging in patients with atypical presentations, rapid clinical progression, or failure to improve despite antimicrobial therapy. Early recognition, targeted antimicrobial therapy, and serial MRI monitoring can lead to favorable outcomes without surgical intervention. Clinicians should remain vigilant for such presentations, particularly in the absence of classic cutaneous features. This case further illustrates the importance of ensuring meningococcal vaccine coverage across all relevant pediatric age groups, reinforcing the public health value of recently updated national immunization programs.
